# Case study: auditory brain responses in a minimally verbal child with autism and cerebral palsy

**DOI:** 10.3389/fnins.2015.00208

**Published:** 2015-06-19

**Authors:** Shu H. Yau, Genevieve McArthur, Nicholas A. Badcock, Jon Brock

**Affiliations:** ^1^ARC Centre of Excellence in Cognition and its Disorders, Macquarie UniversitySydney, Australia; ^2^Department of Cognitive Science, Macquarie UniversitySydney, Australia; ^3^Department of Psychology, Macquarie UniversitySydney, Australia

**Keywords:** autism, autism spectrum disorder, language impairment, magnetoencephalography, event-related potentials, auditory processing, cerebral palsy, Autistic disorder

## Abstract

An estimated 30% of individuals with autism spectrum disorders (ASD) remain minimally verbal into late childhood, but research on cognition and brain function in ASD focuses almost exclusively on those with good or only moderately impaired language. Here we present a case study investigating auditory processing of GM, a nonverbal child with ASD and cerebral palsy. At the age of 8 years, GM was tested using magnetoencephalography (MEG) whilst passively listening to speech sounds and complex tones. Where typically developing children and verbal autistic children all demonstrated similar brain responses to speech and nonspeech sounds, GM produced much stronger responses to nonspeech than speech, particularly in the 65–165 ms (M50/M100) time window post-stimulus onset. GM was retested aged 10 years using electroencephalography (EEG) whilst passively listening to pure tone stimuli. Consistent with her MEG response to complex tones, GM showed an unusually early and strong response to pure tones in her EEG responses. The consistency of the MEG and EEG data in this single case study demonstrate both the potential and the feasibility of these methods in the study of minimally verbal children with ASD. Further research is required to determine whether GM's atypical auditory responses are characteristic of other minimally verbal children with ASD or of other individuals with cerebral palsy.

## Introduction

According to recent estimates, around 30% of individuals with autism spectrum disorders (ASD) remain nonverbal or minimally verbal despite intervention (Coleman, 2000; Mody and Belliveau, 2013; Tager-Flusberg and Kasari, 2013). A significant proportion of these individuals never speak, while others remain at the stage of echolalia or have a limited repertoire of fixed words and phrases that may be communicated through alternative/augmentative communication systems (Kasari et al., 2013). Yet the vast majority of research on cognition and brain function in ASD focuses on high-functioning individuals with age-appropriate or only mildly-impaired language and cognitive abilities. This reflects the practical difficulties of testing these profoundly affected individuals, as well as concerns that results may be compromised by failure to understand task instructions or comply with task demands. However, it is questionable whether insights gained from studies of linguistically able individuals with ASD may be extrapolated to those who are minimally verbal.

To conduct research with minimally verbal children with ASD, it is important to develop valid measures that do not depend upon the ability to understand task instructions or comply with task demands. In principle, neurophysiological techniques such as electroencephalography (EEG) and magnetoencephalography (MEG) are well suited to this purpose (Tager-Flusberg and Kasari, 2013). Electroencephalography reflects electrical activity from populations of synchronously firing neurons (Luck, 2005), while MEG measures the corresponding magnetic fields (Hämäläinen et al., 1993; Hari et al., 2010). Both techniques are safe, noninvasive, and silent, and can provide insights into the neural mechanisms underpinning cognitive function with millisecond precision. Importantly, EEG and MEG responses can often be recorded passively while the participant is engaged in another activity, thereby avoiding concerns about confounding influences of poor task understanding and poor attention.

MEG and EEG offer complementary strengths. MEG has superior spatial resolution because the brain's magnetic fields are not “smeared” or distorted by the brain, scalp, and skull, and are less prone to physiological noise compared to EEG (Hämäläinen et al., 1993; Hari et al., 2010). This allows for cleaner extraction of brain responses that are simpler to interpret. MEG set up is relatively easy and requires no physical contact with sensors, and so is well tolerated by verbal children with ASD (Roberts et al., 2008; Hari et al., 2010; Brock et al., 2013). On the other hand, EEG is much cheaper and more widely available, making it a more realistic tool for large-scale multi-site studies and clinical applications.

Despite their considerable potential, MEG and EEG studies of profoundly affected individuals with ASD are rare. To date, such studies have focussed on auditory processing of simple tone stimuli (Seri et al., 1999; Ferri et al., 2003; Tecchio et al., 2003). Using MEG, Tecchio et al. (2003) tested 8- to 32-year-old autistic individuals with “moderately to severely impaired” verbal communication (according to the Childhood Autism Ratings Scale). Relative to typically developing control participants, they showed a normal M100 response to the onset of tones, but a weak or absent mismatch response to rare sounds in the sequence. In contrast, Ferri et al. (2003) found no evidence of group differences in the mismatch response or subsequent P3a response. Participants were described as having “low functioning autism” and “mental retardation,” but unfortunately no further details were provided regarding their language proficiency or if they were nonverbal.

The current paper adds to this extremely sparse literature on auditory processing in minimally verbal individuals with ASD. We present a case report of GM, a young autistic girl with cerebral palsy who, at the time of writing, has never spoken. When GM was 8 years and 10 months old, we had the opportunity to measure her brain responses to vowels sounds and complex tones using MEG. Two years later, we were able to re-test GM, this time using a novel “gaming” EEG headset that has been adapted for research purposes. Together, the two experiments indicate that GM has a highly unusual pattern of brain responses, characterized by atypically strong responses to nonspeech sounds, but weak responses to speech. This case report demonstrates, we believe, the feasibility and potential of both EEG and MEG for the study of minimally verbal individuals with ASD as well as those with cerebral palsy.

### Background

GM is a young girl with ASD and cerebral palsy. At the time of testing for Experiment 1, she was 8 years and 10 months old. By the time of Experiment 2, she was 10 years and 10 months old. Although she does vocalize, she has never spoken in words, and currently uses an augmentative and alternative communication system on the iPad with prompting from her mother to communicate. She attends a school for children with special needs. Other than her cerebral palsy, GM has no history of brain injury or epilepsy. She has no history of ear infections, and was not on medications at the time of either testing session. Her family speaks Australian English at home.

GM was diagnosed with cerebral palsy (spastic diplegia) aged 18 months. She has global developmental delay and did not walk until after her third birthday. Her mother reports that, as an infant, she had good eye contact and social communication development but lost this at around 18 months. Her diagnosis of DSM-IV Autistic Disorder was conferred by a developmental pediatrician at 59 months (American Psychiatric Association, 1994). Under DSM-5, she would, therefore, automatically qualify for a diagnosis of ASD (American Psychiatric Association, 2013). GM's ASD diagnosis was further supported by her “Lifetime” score of 29 on the Social Communication Questionnaire (SCQ; Rutter et al., 2003), which is well above the threshold of 15 for suspected ASD. Module 1 of the Autism Diagnostic Observation Schedule (Lord et al., 2002) was administered but discontinued because GM showed distress early in the assessment and increased frustration when expected to play. During ADOS administration, she failed to engage in any of the activities, and did not partake in imitation, free play, and reciprocal interaction. While she vocalized sporadically, she did not initiate, engage in, or respond to speech directed at her.

### Cognitive abilities and adaptive behavior

During the testing session for Experiment 1, we attempted to administer a number of standardized tests including the Peabody Picture Vocabulary Test—4th Edition (Dunn and Dunn, 2007) and the Matrices subtest of the Wechsler Intelligence Scale for Children (Wechsler, 2003). Testing using the standard procedures was unsuccessful, largely due to GM's severe communication challenges and her lack of engagement with the tasks. However, GM's mother was able to provide a report from a Clinical Psychologist and Senior Clinical Neuropsychologist of an assessment conducted at age 8 years and 2 months using modified procedures. Relevant sections from the report are reproduced below, with the caveat, noted by the clinicians, that the results of testing may have under-represented GM's true abilities.

“The administration of assessment protocol was adapted due to the severity of [GM's] attention and expressive language difficulties. Task instructions were often repeated and the examiners pointed to relevant stimuli to help [GM] focus. Tasks were selected that allowed [GM] to point to her answer and tasks that required a single word or two word response, that [GM] could type on a computer or her iPad….”

“The nonverbal subtests on the WISC were administered to assess [GM's] level of intellectual functioning… The Block Design subtest could not be administered because of [GM's] motor difficulties… [GM's] visual processing and abstract reasoning ability were found to fall within the ‘extremely low’ range. The results indicated that [GM's] performance/nonverbal skills were consistent with mild to moderate level of intellectual disability….”

“[GM's] understanding of vocabulary was measured with the PPVT… On formal testing, her performance was consistent with a 3–4 year age level….”

“[GM's mother] completed the [Adaptive Behavior Assessment System—2nd Edition] which assesses a child's level of independence in everyday living including the areas of communication, daily and community living skills, social and leisure, functional pre-academics, and motor skills. [GM's] skills overall were in the significantly delayed or ‘extremely low’ range. There was no significant variation evident in her overall level of functioning.”

### Auditory sensory processing

Given the study's focus on auditory processing, GM's mother also completed the Short Sensory Profile (McIntosh et al., 1999), a parent questionnaire that addresses the sensory processing of the child in everyday situations. GM scored within the typical range for the Tactile, Taste/Smell, Movement and Visual/Auditory Sensitivity items. She scored within the Probable Difference range for the Underresponsive/Seeks Sensation and Auditory Filtering items and within the Definite Difference range in the Low Energy/Weak section, which relates to under-responsiveness to vestibular and proprioceptive sensation (Lane et al., 2011). Within the auditory items specifically, she was reported to have never responded negatively to unexpected or loud noises, nor to hold hands over ears, or have trouble completing tasks when the radio is on. However, she was reported to be occasionally distracted or have trouble functioning in noisy environments. Further, she was reported to not hear people, not respond to her name being called, and have difficulties with attention.

Written informed consent was obtained from the mother of the patient for publication of this Case report. A copy of the written consent is available for review by the Editor-in-Chief of this journal.

## Experiment 1

In Experiment 1, we used MEG to investigate GM's brain responses to speech and nonspeech sounds. Procedures for this experiment and Experiment 2 were approved by the Macquarie University Human Research Ethics Committee. Written consent was obtained from parents of all participants, who were given a modest amount of money, a small prize, and a certificate for their participation.

### Participants

At the time of testing, GM was 8 years and 10 months old. Her brain responses were compared to those of 18 typically developing (TD) children (15 boys) and 13 verbal children with ASD (11 boys), aged between 6 and 14 years, who were tested as part of a separate study (Yau et al., in press). All children spoke English as a first language and had normal hearing as determined using an Otovation Amplitude T3 series audiometer.

All children with ASD had reports from psychologists or pediatricians confirming their DSM-IV (American Psychiatric Association, 1994) and/or ICD-10 (World Health Organisation, 1992) diagnosis of an ASD. In addition, they all scored above the Autism cut-off on the SCQ. All children in the ASD group (“Verbal ASD”) had phrase speech, although performance on standardized language assessments varied widely, as shown in Table 1. Typically developing children scored below the Autism cut-off on the SCQ, and reported no history of brain injury, ASD, language impairment, or developmental disorders in their family.

**Table 1:** **Characteristics of children in the verbal autism spectrum disorders (ASD) and typically developing (TD) comparison groups**.

	**ASD (*N* = 13)**		**TD (*N* = 18)**	
	***M* (*SD*)**	***Range***	***M* (*SD*)**	***Range***
Age (years)	10.82 (1.72)	7.75–13.25	10.02 (2.39)	6.67–14.58
Matrices (WISC-IV)^a^	8.71 (3.52)	4–14	12.44 (2.28)	9–16
Receptive vocabulary (PPVT-II)^b^	98.00 (23.96)	61–160	121.39 (15.45)	92–167
Receptive grammar (TROG-II)^b^	85.29 (18.49)	55–111	106.67 (8.94)	85–123
Sentence repetition (CELF)^a^	5.71 (3.69)	1–11	10.72 (2.27)	7–15
Social communication questionnaire	24.57 (6.43)	17–37	2.33 (1.82)	0–6

*Participants were assessed on the Matrices subtest of the Wechsler Intelligence Scale for Children (Wechsler, 2003), the Peabody Picture Vocabulary Scale (PPVT-IV; Dunn and Dunn, 2007), the Test for Reception of Grammar (TROG-II; Bishop, 2003), and the sentence repetition subtest of the Clinical Evaluation of Language Fundamentals (Semel et al., 1987). Parents completed the Social Communication Questionnaire (Rutter et al., 2003)*.

a *Scaled scores with population means and standard deviations of 10 and 3*.

b *Standard scores with population means and standard deviations of 100 and 15*.

### Stimuli

Stimuli were 200-ms long with 5-ms ramps at the start and end to avoid clicks and distortions to the sounds. The speech stimulus was a natural sounding English vowel /a/ (McArthur et al., 2009). The nonspeech stimulus was a complex tone created using Adobe Audition to match the first three formants of the speech sound (see Table 2 for stimuli characteristics). The main difference between the two sounds was the presence of a fundamental frequency (F0) in the speech stimuli, which gave the speech sounds their “speechiness.”

**Table 2:** **Speech and nonspeech stimuli acoustic parameters**.

**Formant**	**Speech**			**Nonspeech**		
	**Hertz**	**Milliseconds**	**Bandwidth**	**Hertz**	**Milliseconds**	**Bandwidth**
F0	106–119	5–20	NA	NA	NA	NA
	120	25–80	NA	NA	NA	NA
	119–179	85–200	NA	NA	NA	NA
F1	700	5–200	70	700	5–200	NA
F2	1560	5–200	130	1560	5–200	NA
F3	2430	5–200	320	2430	5–200	NA

Stimuli were presented binaurally at 75 dB SPL via earphones attached to rubber air tubes (Model ER-30, Etymotic Research Inc., Elk Grove Village, IL). Children were presented eight blocks of 100 speech stimuli interleaved with eight blocks of 100 nonspeech stimuli. The stimulus onset asynchrony (SOA) was jittered between 900 and 1100 ms. The stimuli were presented in an oddball paradigm originally designed to elicit a mismatch field. Each block of 100 sounds included 85 frequently occurring “standard” sounds and 15 rarely occurring “deviant” sounds (a 10% increase in the frequency of F1, F2, and F3 relative to the standard sound). However, like other researchers, we found that the mismatch response was not reliably elicited at the individual level (Kurtzberg et al., 1995; Uwer and von Suchodoletz, 2000; McArthur et al., 2003; Bishop, 2007; Mahajan and McArthur, 2012). Thus, following past research, our analyses focused on the obligatory brain responses to the onset of the standard stimuli (McArthur and Bishop, 2005; Whitehouse and Bishop, 2008).

### MEG recording

MEG data were recorded using 160 coaxial first-order gradiometers with a 50 mm baseline (Model PQ1160R-N2, KIT, Kanazawa, Japan; Kado et al., 1999; Uehara et al., 2003). MEG data were acquired with a sampling rate of 1000 Hz and filter bandpass of 0.03–200 Hz. Prior to MEG recording, each child was fitted with an elasticized cap containing five marker coils. The positions of the coils and the shape of the participant's head were measured with a pen digitizer (Polhemus Fastrack, Colchester, VT). Head position was measured with the marker coils before and after each MEG recording, and children were visually monitored for head movements. If the authors detected movement from the child, data recording for that block was aborted and marker coils re-measured. Children who exceeded head-movement of 5 mm were excluded from further analyses. During the recording, participants watched a silent subtitled DVD of their choice projected on a screen on the ceiling of the MEG room while lying on a comfortable bed inside the magnetically shielded room.

### MEG data processing

MEG data were processed using BESA 6.0 software (MEGIS Software GmbH, Grafelfing, Germany). The data were filtered between 0.1 and 30 Hz, epoched from −100 ms pre-stimulus onset to 500 ms post-stimulus onset, and baseline corrected from −100 to 0 ms. Epochs with gradient artifact (including blinks and eye-movements) greater than 5336 fT/cm were identified using the artifact-rejection tool in BESA, and excluded from further analysis. All participants had at least 75% artifact-free epochs for each condition. On average, there were 542 accepted epochs for speech sounds and 538 for nonspeech sounds in the control group. For GM, there were 448 accepted epochs for speech sounds and 494 for nonspeech sounds.

Data were first analyzed at the sensor level by computing the Global Field Power (GFP, Lehmann and Skrandies, 1980). This involved transforming the speech and nonspeech waveforms for each of the 160 sensors to absolute values and then averaging across the 160 channels to obtain a whole head response (cf. Kasai et al., 2005). This procedure avoids bias that may arise from picking a group of channels and complements analyses conducted in source space. Magnetic GFP also strongly corresponds with fitted dipoles in terms of strength and latency, and is considered a good representation of underlying brain activity from the sources (Kasai et al., 2002, 2003).

Data were also analyzed in source space using BESA 6.0. For each participant, we first averaged the sensor data across the speech and nonspeech conditions. Two dipoles were initially placed in bilateral Heschl's gyrus (according to the template brain) and then fitted freely (location and orientation), subject to the constraint that their locations remained symmetrical. For most participants, dipoles were fitted and optimized to the 80–110 ms window, corresponding to each child's M50/M100 response. However, in some cases, it was necessary to extend the time window down to 70 ms or up to 160 ms to more accurately account for latency delays in younger children or those with maturing waveforms (cf. Oram Cardy et al., 2004). Separate speech and nonspeech source waveforms were then extracted from the left and right hemisphere dipoles.

## Results and discussion

**Figure 1:**
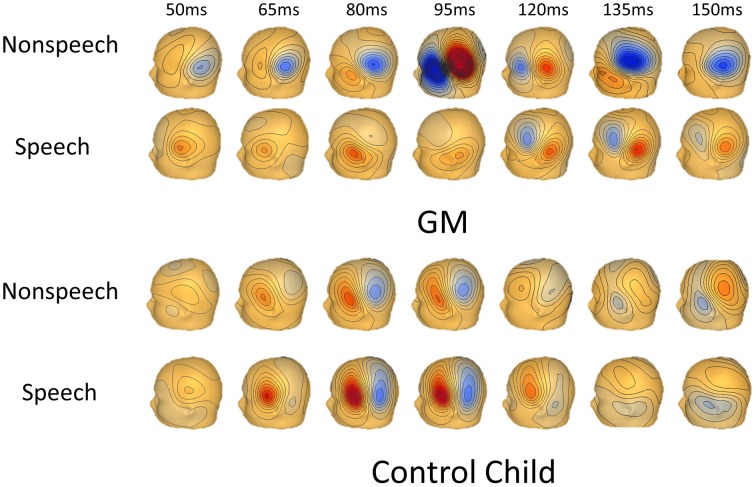
**Timeline of brain activity to speech and nonspeech stimuli for GM and an age-matched child**. Timeline of magnetic flux activity showing obligatory brain activity (auditory M50/M100) from left hemisphere sensors to speech and nonspeech stimuli. The top two rows are GM and the bottom two rows are of an age-matched typically developing child to nonspeech and speech stimuli.

Figure 1 shows a timeline of GM's magnetic flux map for speech and nonspeech responses. Note that compared to the age-matched typically developing child in Figure 1, GM's response to nonspeech was much earlier and larger than her response to speech.

**Figure 2:**
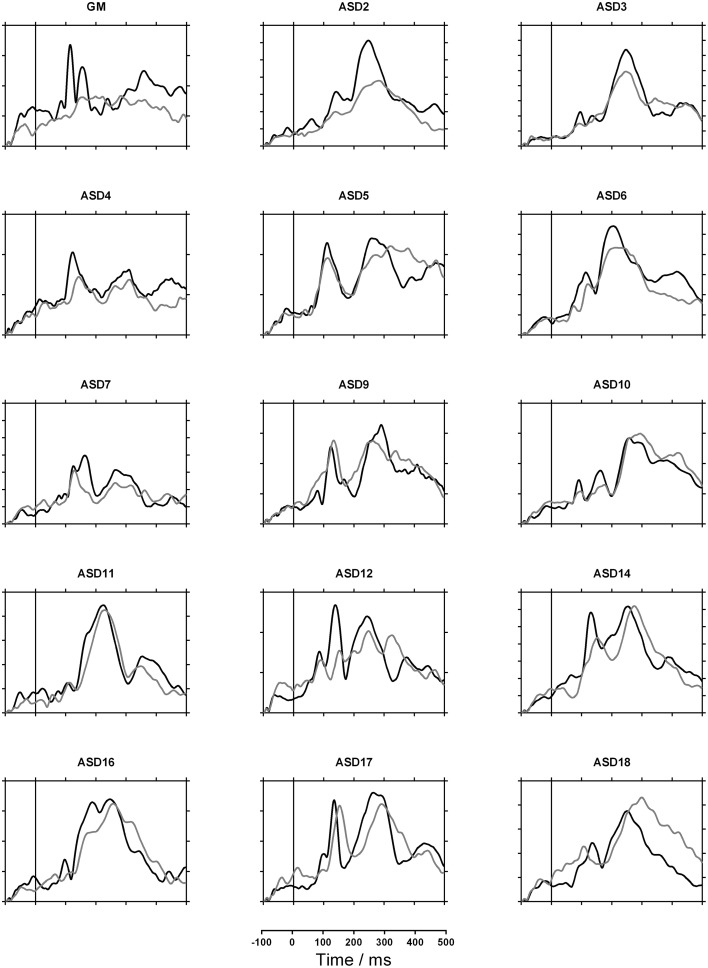
**MEG Sensor waveforms for GM and all verbal children with autism spectrum disorder (ASD)**. Gray lines indicate response to speech and black lines indicate nonspeech response. Each tick on the vertical axis represents 10 femtoTesla.

**Figure 3:**
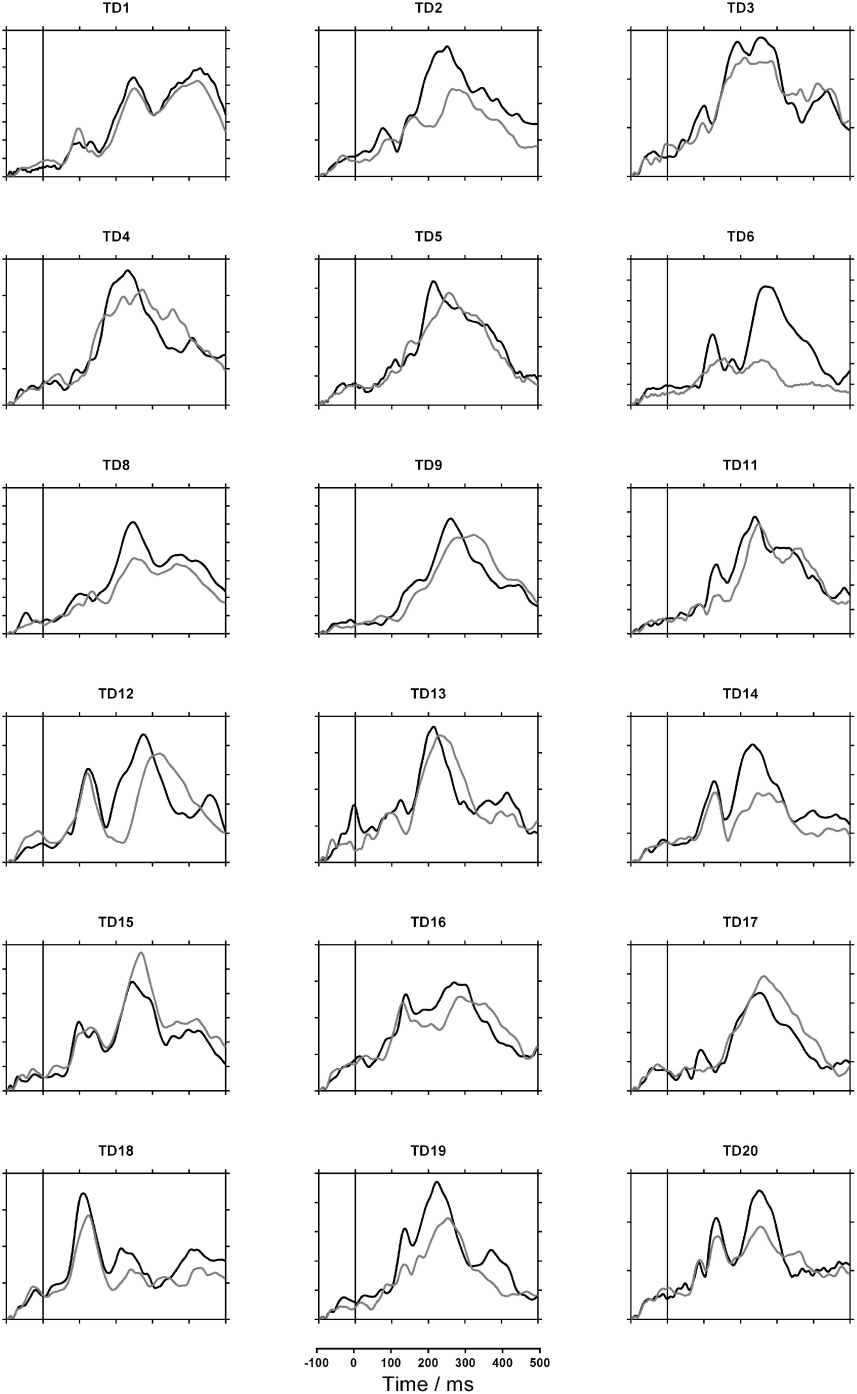
**MEG sensor waveforms for typically developing (TD) children**. Gray lines indicate response to speech; black lines indicate nonspeech response. Each tick on the vertical axis represents 10 femtoTesla.

Figures 2, 3 show each participant's sensor waveforms to speech and nonspeech sounds. Again, there was a discrepancy between GM's double-peaked response to nonspeech stimuli and her virtually flat response to speech. In contrast, the other participants showed similar responses to speech and nonspeech stimuli. Note however, that the participants differed widely in both the morphology of the waveforms and their overall magnitude. While this may partly reflect differences in brain activity, it may also depend on the child's position in the MEG helmet and the size of their heads.

To quantify the similarity between each participant's speech response and their own nonspeech responses, we used intra-class correlations (ICCs), which were Fisher-z transformed to improve linearity for parametric statistics (cf. Bishop and McArthur, 2004, 2005). Initially, we included the whole epoch in the ICC calculations (0–500 ms). In addition, we also considered a narrower 65–165 ms window, which incorporated the obligatory M50 and M100 responses (see Yau et al., in press).

We compared GM's ICCs to those of children in the TD and ASD comparison groups using SingLims, a statistical program widely used in neuropsychological case studies (Crawford et al., 2010). The SingLims approach assumes the comparison participants to be a representative sample of the population, and uses modified t-tests to estimate the “abnormality or rarity” of a case's scores and the percentile ranking of the case (i.e., the percentage of the control population exhibiting a lower score than the case). Tables 3, 4 show the SingLims test results, and point and interval estimates of effect size and abnormality for GM's scores, compared to the TD and ASD comparison groups respectively. GM's ICCs were significantly lower than both control groups for both the 65–165 ms and 0–500 ms time periods, in each case placing her in the bottom 5% of the population.

**Table 3:** **Outcome of SingLims analysis comparing GM to the typically developing (TD) control group**.

	**TD group**			**GM**	**GM vs. TD**		**Percentile**		**Effect size**	
	***N***	**Mean**	***SD***		***t***	***p***	**Point**	**95% CI**	**Point**	**95% CI**
**SENSOR WAVEFORMS (GLOBAL FIELD POWER)**										
0–500 ms	18	1.11	0.39	−0.11	−3.04	0.00	0.37	0.00–2.38	−3.13	−4.26 to −1.98
65–165 ms	18	0.73	0.48	−0.16	−1.81	0.04	4.44	0.45–14.17	−1.85	−2.62 to −1.07
**SOURCE WAVEFORMS (65–165 ms)**													
Left	18	0.45	0.52	−0.66	−2.08	0.03	2.66	0.15–10.08	−2.13	−2.97 to −1.28
Right	18	0.54	0.63	0.13	−0.63	0.27	26.74	12.44–44.73	−0.65	−1.15 to −0.13

*Percentile point shows percentage of the control population exhibiting a lower score than GM. The 95% confidence interval (95% CI) denotes the certainty of the point percentile (Crawford et al., 2010), or the certainty of the rarity of GM's scores. The 95% CI in effect size denotes the certainty or credibility of the effect size of the differences between GM and controls*.

**Table 4:** **Outcome of SingLims analysis comparing GM to the autism spectrum disorders (ASD) control group**.

	**ASD group**			**GM**	**GM vs. ASD**		**Percentile**		**Effect size**	
**Measure**	***N***	**Mean**	***SD***		***t***	***p***	**Point**	**95% CI**	**Point**	**95% CI**
**SENSOR WAVEFORMS (GLOBAL FIELD POWER)**													
0–500 ms	14	0.98	0.54	−0.11	−1.95	0.03	3.65	0.16–14.06	−2.02	−2.94 to −1.08
65–165 ms	14	0.58	0.35	−0.16	−2.04	0.03	3.09	0.11–12.65	−2.11	−3.06 to −1.14
**SOURCE WAVEFORMS (65–165 ms)**										
Left	14	0.70	0.50	−0.66	−2.63	0.01	1.04	0.00–6.04	−2.72	−3.87 to −1.55
Right	14	0.55	0.50	0.13	−0.81	0.22	21.58	7.47–41.49	−0.84	−1.44 to −0.21

*Percentile point shows percentage of the control population exhibiting a lower score than GM. The 95% confidence interval (95% CI) denotes the certainty of this point percentile (Crawford et al., 2010), or the certainty of the rarity of GM's scores. The 95% CI in effect size denotes the certainty or credibility of the effect size of the differences between GM and controls*.

**Figure 4:**
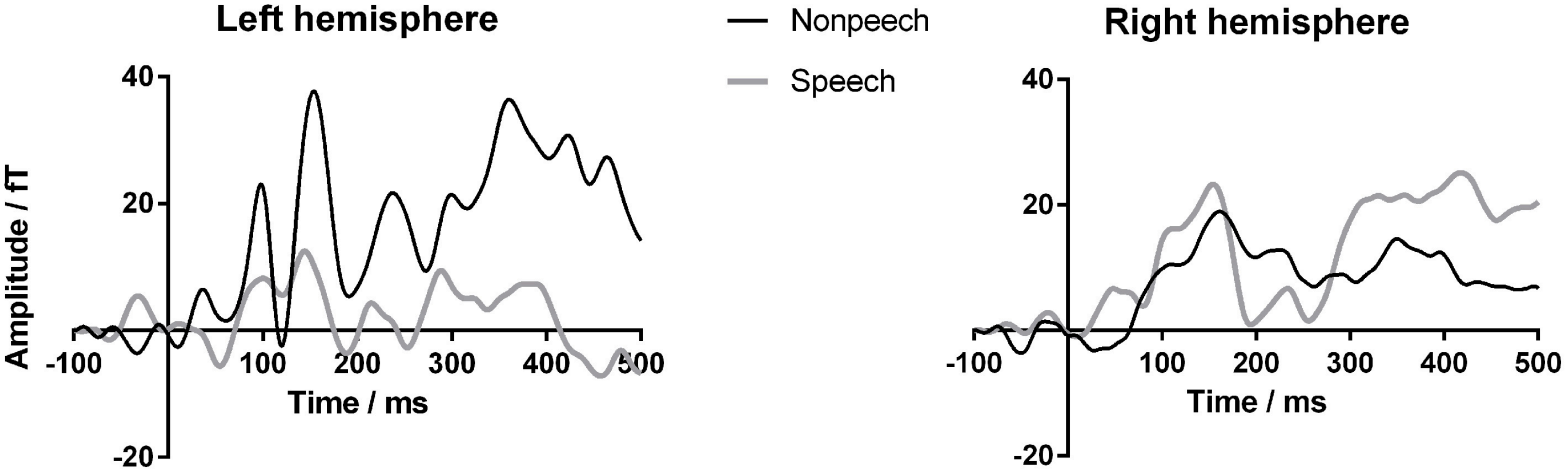
**GM's source waveforms for speech and nonspeech stimuli from Experiment 1**. Magnetoencephalography (MEG) source waveforms from left and right hemisphere sources approximating auditory cortex from Experiment 1. Gray lines indicate response to speech; black lines indicate nonspeech response. Vertical axis represents amplitude in femtoTesla.

Figure 4 shows the results of the source analysis for GM. It suggests that the striking differences between GM's speech and nonspeech sensor waveforms originate from the left hemisphere. As for the sensor analysis, we calculated Fisher z-transformed ICCs to index the similarity of each child's nonspeech and speech dipole waveforms, for left and right hemisphere sources. As the dipoles used for source extraction were oriented to the M50/M100 response, we only report ICCs for the corresponding 65–165 ms window. SingLims analyses (Tables 3, 4) show that GM had significantly reduced ICCs for the left hemisphere, again placing her in the bottom 5% of the population relative to both control groups. In contrast, her right hemisphere responses were within the normal range.

To summarize, GM's MEG recordings were highly atypical. In particular, she showed a striking dissociation between her M50/M100 responses to speech and to nonspeech sounds. This appeared to originate in her left auditory cortex and was not shown by any of the typically developing or autistic children we tested.

It is important to consider the possibility that GM's atypical recordings may be artefactual. Of particular concern is the possibility that GM may have moved more than other participants during the recording session. The KIT MEG system does not currently incorporate online motion tracking. However, during MEG testing, all participants, including GM, were monitored carefully for head-motion, with strict data acquisition and exclusionary criteria applied for motion (see MEG Recording). Moreover, they were lying in a supine position that helps support the head and reduce unwanted movements during recording (Herdman and Cheyne, 2009). Finally, and perhaps most importantly, it is highly unlikely that excessive motion could have given rise to the specific pattern of responses we have reported. We would not expect motion to affect responses to speech and nonspeech differentially or to result in exaggerated hemispheric asymmetries. Nor would we expect artifacts to result in a *clearer* response to nonspeech than that found in controls.

## Experiment 2

Two years after the initial recording session, we had the opportunity to re-test GM as part of a second ongoing study, the aim of which was to validate a lightweight “wireless gaming” EEG system as a research tool for use with typically developing children (Badcock et al., 2015). If the findings from Experiment 1 were a genuine reflection of atypical brain responses, we expected to find similar atypicalities in GM's EEG recordings. Replicating our findings from Experiment 1 would also provide preliminary evidence for the suitability of the gaming EEG system for the assessment of minimally verbal children with ASD.

### Participants

At the time of testing for Experiment 2, GM was 10 years and 10 months old. Her auditory brain responses to nonspeech sounds were compared to those of 21 TD children (11 females, 10 males) aged between 6 and 12 years, tested using the same procedures as part of a validation study for the EEG system. The mean age of TD participants was 9.23 years (*SD* = 1.78). Participants had normal hearing and vision, and no history of developmental disorders or epilepsy.

### Stimuli

Stimuli were standard tones (*n* = 566, 175-ms 1000-Hz pure tones with a 10-ms rise and fall time; 85% of trials) and deviant tones (*n* = 100, 175-ms 1200-Hz pure tones with a 10-ms rise and fall time; 15% of trials), separated by a jittered SOA of 900–1100 ms. Tones were presented binaurally at a comfortable listening volume through speakers. Participants in the TD group heard 666 tones in a single block. Due to concerns about potential movement artifacts, GM was presented with a second block of 666 trials after a short break.

### EEG recording and analysis

Participants were seated in a comfortable chair and watched a silent video whilst ignoring the tones. Auditory brain responses were measured using an Emotiv EPOC gaming EEG system that has previously been validated against a research-grade Neuroscan EEG system (Badcock et al., 2013). The sensors in the headset were adjusted on the head until suitable connectivity was achieved as indicated by the TestBench software, which adds a small modulation to the feedforward signal, and measures the size of the signal back from each channel. The testing procedure took 10–15 min.

The Emotiv EEG system uses gold-plated contact-sensors fixed to flexible plastic arms of a wireless headset. The headset included 16 sites, aligned with the 10–20 system: AF3, F7, F3, FC5, T7, P7, O1, O2, P8, T8, FC6, F4, F8, AF4, M1, and M2. One mastoid (M1) sensor acted as a ground reference point to which the voltage of all other sensors were compared. The other mastoid (M2) was a feed-forward reference that reduced external electrical interference. The signals from the other 14 scalp sites (channels) were high-pass filtered with a 0.16 Hz cut-off, pre-amplified and low-pass filtered at an 83 Hz cut-off. The analog signals were then digitized at 2048 Hz. The digitized signal was filtered using a 5th-order sine notch filter (50–60 Hz) and low-pass filtered and down-sampled to 128 Hz. The effective bandwidth was 0.16–43 Hz.

The Emotiv EEG system was modified to send markers to the EEG to indicate the onset of each stimulus (Thie et al., 2012). This was achieved using a custom-made transmitter that converted the onset and offset of each tone into a positive and negative electrical signal. These signals were transmitted into the O1 and O2 channels using an infrared triggering system. The positive and negative spikes in the O1 and O2 EEGs were processed offline in MATLAB. A between-channels difference greater than 50 mV was coded as a stimulus onset or offset. The event marker had at a constant time interval (20 ms delay of the transmitter module) prior to the point of positive and negative signal cross-over. Stimulus markers were recombined with the EEG data.

The resultant EEG was processed offline using EEGLAB version 11.0.5.4b (Delorme and Makeig, 2004). The EEG in each channel was bandpass filtered from 0.1 to 30 Hz, and then divided into epochs that started 102 ms before the onset of each stimulus and ended 500 ms after the onset of the same stimulus. Each epoch was baseline corrected from -102 to 0 ms. Epochs with absolute values greater than 150 uV were rejected.

To maximize the amount of useful data, we collapsed across tone types (standard and deviant). For GM, this resulted in a total of 220 accepted epochs across her two blocks of recordings. For control participants, there were many more acceptable trials (mean = 617, *SD* = 42 for a single block), but in order to equate GM and the controls for data quality, for each TD participant, we randomly sampled 220 trials (including standards and deviants). For each participant, we then averaged the 220 epochs to create an auditory ERP.

## Results and discussion

**Figure 5:**
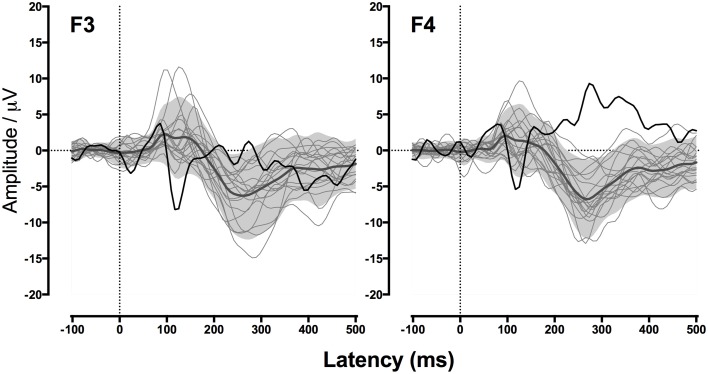
**GM's waveforms for nonspeech stimuli from Experiment 2**. Event-related potentials (ERPs) to nonspeech sounds measured from frontal electrodes F3 (left) and F4 (right). Black line shows GM's response. Gray region indicates the average response of children with typical development (TD) for ±1.64 SD (considered the “normal” range). Light gray lines show responses of individual TD children.

Figure 5 shows GM's responses recorded from the two electrodes, F3 (left frontal) and F4 (right frontal), that produced the clearest response in the TD control participants. Consistent with her atypically large MEG response to nonspeech stimuli in Experiment 1, GM showed a strikingly strong and early response to the tone stimuli, particularly for the left frontal electrode. This was clearly outside the range of any of the TD control participants. Thus, GM's unusually large brain response to nonspeech stimuli appears to be a stable and replicable characteristic of her cortical response to a range of nonspeech stimuli.

## General discussion

Minimally verbal individuals represent a significant proportion of the autistic population and yet are typically excluded from research on cognition and brain function. In the current study, we used MEG and EEG to measure the brain responses to auditory stimuli of a minimally verbal child with ASD. The initial MEG study in Experiment 1 revealed a striking dissociation between her auditory sensory encoding of speech and nonspeech sounds. Specifically, GM had relatively strong and early responses to nonspeech, but unusually weak responses to speech sounds. MEG source analysis suggested that these differences arose in her left hemisphere. We were able to demonstrate statistically that this discrepancy between speech and nonspeech stimuli was highly unusual. Whether compared to typically developing children or other verbal children with ASD, GM's response similarity for speech and nonspeech fell into the bottom 5% of the population.

In Experiment 2, we replicated the finding that GM shows unusually strong response to nonspeech stimuli. This was observed despite the fact she was tested 2 years after Experiment 1 using a different neurophysiological technique (EEG rather than MEG), in a different environment, using different stimuli (pure tones rather than complex tones), as well as a different control sample. This successful replication indicates that GM's atypical responses to nonspeech sounds are genuine and not merely a consequence of methodological artifacts.

GM's atypical responses to nonspeech sounds in both experiments might be considered a neural correlate of atypical auditory processing that is widely reported amongst individuals with ASD (Boddaert et al., 2004; Gervais et al., 2004). Autobiographical accounts of individuals with ASD often include descriptions of atypical sensory experiences, particularly in relation to sounds (Grandin and Scariano, 1986; Bettison, 1996; Reynolds and Lane, 2008; Ben-Sasson et al., 2009). These accounts are supported by parental reports, clinical observations, and enhanced performance on certain psychoacoustic tests (Bonnel et al., 2003, 2010; Tomchek and Dunn, 2007; Heaton et al., 2008; Jones et al., 2009). Surprisingly, then, GM appears to show little evidence of hyper-responsiveness to auditory stimuli in everyday life, as documented by her mother's responses on the Short Sensory Profile. Given that GM is nonverbal, we were unable to obtain a self-report of her sensory experiences. Thus, it remains an open question what the subjective experience of her atypical cortical responses might be.

Clearly, the other intriguing aspect of GM's data is her attenuated response to speech stimuli in the MEG experiment. One interpretation is that GM's brain “switches off” to speech stimuli. This would be consistent with the theories of social deficit or an impairment in social motivation and cognition in ASD (Dawson et al., 1998, 2004; Klin, 2003; Chevallier et al., 2012) and with previous ERP studies suggesting that children with ASD show a difference in the attentional orienting to speech and nonspeech sounds, particularly when they are not explicitly required to attend to the sounds (Ceponienë et al., 2003; Lepistö et al., 2005, 2006; Whitehouse and Bishop, 2008). However, previous studies have focused on the later mismatch negativity and P3 components of the auditory ERP, whereas the striking differences between speech and nonspeech in GM's brain responses were apparent much earlier in the waveform, during the “obligatory” M50/M100 components. This suggests that GM's differential response to speech and nonspeech sounds reflects a bottom-up mechanism in her brain's sensitivity to the acoustic differences between the two stimuli.

The major difference between the speech and nonspeech stimuli is the presence of the fundamental frequency (F0) in the speech stimuli. This serves to give a sound its “speechness” and provides pitch cues for conveying linguistic and emotional prosody as well as information about speaker identity (see McCann and Peppe, 2003; Peppé et al., 2007 for review). Perhaps most importantly, the fundamental frequency also provides a vital cue for segregating speech from background noise in natural listening environments (e.g., Bronkhorst, 2000). Thus, a neural impairment affecting the processing of the fundamental frequency might be expected to have profound implications for the development of speech perception.

It is important to note that GM also has a diagnosis of cerebral palsy, which sets her apart from other minimally-verbal autistic children. The nature of the relationship between ASD and cerebral palsy is unclear and difficult to tease apart (Zwaigenbaum, 2014). Although the incidence of ASD is considerably higher amongst individuals with cerebral palsy (approximately 6%; Christensen et al., 2014) than it is in the general population, the majority of individuals with cerebral palsy do not meet ASD criteria. Likewise, speech and language abilities are affected in the majority of individuals with cerebral palsy, but the complete absence of speech is relatively rare (Odding et al., 2006).

## Concluding remarks

The current case report represents a starting point for investigating the potential causes of severe language impairment that affect many individuals on the autism spectrum. However, GM is obviously an unusual case and, at this stage, it is unclear whether or not her atypical brain responses might generalize either to other minimally verbal children with ASD or to those with cerebral palsy. Nonetheless, the current study stands as an important proof of concept, demonstrating that it is possible in practice to measure brain responses to different auditory stimuli, using both MEG and EEG, from minimally verbal children with ASD. Future studies can take advantage of the complementary strengths of these two techniques and begin to answer vital questions pertaining to cognition and brain function within this much-neglected subgroup of the ASD population.

## Author contributions

SY and JB contributed to the conception, design, acquisition of data, analysis, interpretation, drafting and revising the manuscript. GM contributed to data interpretation, drafting and revising the manuscript. NB contributed to the conception, design, acquisition of EEG data, EEG analysis, interpretation, and drafting the manuscript. All authors read and approved the final manuscript and have given final approval of the version to be published.

### Conflict of interest statement

The authors declare that the research was conducted in the absence of any commercial or financial relationships that could be construed as a potential conflict of interest.
